# Immersive Virtual Reality–Supported Cognitive-Behavioral Therapy for Patients With Mild to Borderline Intellectual Disabilities and Substance Use Disorders: Two Exploratory Studies

**DOI:** 10.2196/82601

**Published:** 2026-05-04

**Authors:** Samantha Murray, Simon Langener, Hanneke Kip, Randy Klaassen, Saskia Marion Kelders, Dirk Heylen, Joanne VanDerNagel

**Affiliations:** 1 Behavioural Management and Social sciences, Psychology Health and Technology, University of Twente, Drienerlolaan 5, Enschede, 7522 NB, The Netherlands, 31 534896027; 2 Department of Research, Transfore, Deventer, The Netherlands; 3 Human Media Interaction, University of Twente, Enschede, The Netherlands; 4 Centre for Addiction and Intellectual Disability, Tactus Addiction Care, Enschede, The Netherlands; 5 Nijmegen Institute for Scientist-Practitioners in Addiction, Radboud University, Nijmegen, The Netherlands

**Keywords:** immersive virtual reality, cognitive behavioral therapy, substance use disorder, alcohol use disorder, nicotine dependence, mild intellectual disability, borderline intellectual functioning, craving, relapse prevention, user-centered design

## Abstract

**Background:**

Substance use disorders (SUDs) are prevalent and characterized by high relapse rates. Individuals with mild to borderline intellectual disability (MBID) are more likely to develop SUDs and face barriers within treatment related to difficulties they experience with abstract thinking, verbal skills, and generalizing learned strategies to real-world contexts. Therefore, experiential, context-rich approaches are needed that reduce reliance on retrospective verbal reflection, support in-context identification of triggers, and allow the rehearsal of coping responses. Immersive virtual reality (IVR) may provide realistic, safe environments where patients with SUD and MBID can practice cognitive and behavioral skills with visual and practice-oriented materials.

**Objective:**

This study aimed to generate design input for the development and clinical integration of IVR-supported therapy for individuals with MBID and SUD. Specifically, Study 1 explored alcohol-related triggers in patients with alcohol use disorder, whereas Study 2 examined the feasibility and acceptability of practicing nicotine-related coping strategies in patients with nicotine dependence (ND).

**Methods:**

Two explorative studies were conducted at an inpatient clinic for patients with MBID and SUD in the Netherlands. Study 1 included 10 adults with alcohol use disorder and MBID who participated in interviews to determine relevant risk situations, triggers, and therapeutic goals for IVR-cognitive behavioral therapy (CBT). Study 2 included 10 adults with MBID and nicotine dependence who practiced coping strategies within an existing IVR featuring craving-inducing and craving-reduction scenarios. A multiple-method approach was used to gather input for IVR-CBT development and to explore feasibility and acceptability (user evaluation interviews, the Questionnaire of Smoking Urges, and Visual Analog Scale ratings).

**Results:**

In study 1, we identified high-risk situations, including at-home routines (eg, sitting on the couch watching football), supermarkets (eg, confrontation with alcohol and advertisements), social gatherings (eg, invitations and peer pressure), and being outside or traveling (eg, public transport or passing alcohol-related places). Triggers clustered into multisensory cues (eg, seeing or smelling alcohol), social influences (peer pressure and interpersonal conflict), affective states (tension, distress, boredom, or euphoria), and personal habits (eg, rewarding oneself or associations with money). Participants expressed interest in using IVR to identify triggers, discuss affective states, and train refusal skills. In study 2, IVR elicited nicotine craving, which increased during cue exposure and decreased during tutorial and coping phases. The coping elements embedded in IVR included relaxation (eg, mindfulness or breathing exercises), distraction (eg, virtual pets and interactive games), and physical activity (eg, walking or sports).

**Conclusions:**

IVR-CBT elements appear feasible and acceptable in inpatient MBID care with appropriate support. Findings provide patient-derived design insights for integrating trigger identification and coping rehearsal within IVR. Future work should use an iterative, user-centered design approach based on validated CBT-related techniques (eg, functional analysis or coping, or skills training) and compare IVR-CBT with CBT as usual to understand benefits and risks for patients and therapists.

## Introduction

### Background

Substance use disorders (SUDs) are highly prevalent and complex psychiatric conditions with a high disease burden [1]. Individuals with mild to borderline intellectual disabilities (MBID) are at an increased risk for developing SUDs [2,3]. MBID covers both persons with mild intellectual disability (MID) and borderline intellectual functioning (BIF) [4], and in the Dutch clinical context, it is defined by an IQ between 50 and 85, accompanied by limitations in adaptive functioning with onset during the developmental period. Individuals with MBID often experience cognitive limitations that affect comprehension, memory, and problem-solving abilities, as well as difficulties in understanding the consequences of substance use, which can increase their susceptibility to SUDs and reduce the efficacy of treatment as usual [2]. Additionally, the prevalence of SUDs among persons with MBID is exacerbated by social vulnerabilities, such as problems with social skills [5], as well as social isolation, trauma, and substance use as a form of self-medication against negative life events [6].

Despite the availability of various psychotherapeutic and pharmacological treatments, maintaining long-term recovery in individuals with MBID can be challenging. As reported by the National Institute on Drug Abuse (2020), relapse rates in the general population who were treated for SUDs vary between 40 and 60 percent. Although specific relapse data for individuals with MBID are limited, relapse rates in this group might be even higher, given the challenges in assessment and standard treatment they may encounter [7,8]. For instance, most individuals with MBID experience communication barriers as they struggle to verbalize their thoughts, emotions, behaviors, and everyday risk situations. Moreover, barriers in skill application are present, as they often experience difficulties with applying newly learned skills from treatment in real-world risky situations [8]. These barriers in communication and skill application can make it more difficult to engage in therapeutic processes such as cognitive behavioral therapy (CBT) that rely on reflective, language-based approaches, which require cognitive and adaptive skills. Although tailored CBT protocols for the dual diagnosis of MBID and SUDs are available in the Netherlands [8,9], they still require patients to remember and reflect on their own behavior within treatment when discussing high-risk situations. Furthermore, generalizing and applying the newly learned coping skills in the real world remains difficult [8]. Consequently, there is a need for experiential, context-rich treatment approaches that reduce the reliance on abstract verbal processing and support in-context rehearsal of coping responses.

### From Virtual Reality Exposure Therapy to Immersive Virtual Reality–Based Cognitive Behavioral Therapy

Immersive virtual reality (IVR) offers a promising approach to address the cognitive barriers that people with MBID experience within standard treatments that are based on the principles of CBT. IVR can be described as a computer-generated simulation of a 3D setting by using special electronic equipment [10]. Typically, head-mounted displays (HMDs) are used to immerse the user into a virtual environment (VE). Previous research suggests that IVR might lower learning obstacles by making experiences and training abstract concepts and interrelationships more explicit, understandable, and trainable, thereby improving comprehension for people who struggle with abstract thinking, such as those with MBID [11-13]. This approach eliminates the requirement for disembedded thinking, which refers to the capacity to grasp concepts outside of immediate and physical contexts. This is a skill that individuals with MBID find challenging [14]. Moreover, IVR can facilitate active learning rather than passively receiving information, thus reducing the reliance on abstract reasoning and verbal instructions while fostering skill acquisition [14]. This implies that learning becomes an engaging experience that does not solely rely on a language-based experience. Instead, IVR facilitates skill development through experiential learning, allowing individuals to learn by doing in a safe, controlled setting and acquire new skills without solely relying on language-based instructions [15,16].

Various studies have shown that VEs can elicit realistic (physical) reactions and behaviors in patients with SUD [10,17,18], including tension and substance cravings [13]. Virtual reality exposure therapies (VRET) have been developed based on these “natural” reactions to stimuli in IVR, for example, within the domain of anxiety disorders [19]. Following this initial success, research has also explored possible uses of VRET for SUDs, drawn by its ecological validity in realistically eliciting cue reactivity, such as drug craving and psychophysiological reactions (eg, increased heart rate and sweating) to stimuli associated with substance use [20].

However, the effectiveness of VRET for SUDs remains questionable. While pilot studies using VRET for SUDs led to cue reactivity and participant satisfaction, as well as expectations of clinical benefit, a 2021 review indicates that this has not yet been demonstrated in research on clinical effectiveness [21]. This is supported by an earlier review from Segawa et al [22], who stated that although IVR is effective at eliciting craving, VRET treatment outcomes vary. One hypothesis is that exposure in the context of addiction does not lead to extinction but rather reactivates a pathologically disturbed reward system, unlike anxiety disorders. As a result, VRET might even increase the risk of relapse [23], which is consistent with the lack of effectiveness of regular exposure therapies for SUDs [24]. Taken together, the evidence at the time of this writing indicates that VRET is insufficient for lasting change and that it may be essential to integrate, for example, CBT techniques in IVR interventions to actively understand triggers and train coping behaviors. This approach aligns with a literature review by Taubin et al [25], who found that IVR protocols integrating mindfulness practice, cognitive reappraisal, or other emotion-regulation tasks alongside IVR were more likely to achieve clinically meaningful effects on substance-use outcomes. Experimental data support this conclusion; in a randomized study, smokers who actively crushed virtual cigarettes, an IVR task that requires users to enact an immediate coping response, achieved significantly higher abstinence rates than smokers who performed a neutral control task [26]. These findings indicate that active rehearsal of coping strategies rather than exposure alone is essential for therapeutic benefits. Accordingly, an IVR protocol used in an active learning format that engages patients in practicing coping responses to trigger-related craving may positively influence internal biases and promote behavior change.

Building on these insights, embedding active learning tasks into an IVR can lead to interactive, user-centered interventions that are more compatible with the needs of individuals with MBID and SUDs. IVR-CBT for SUDs allows patients to safely confront triggers by exploring maladaptive thought patterns and gradually exposing them to drug-craving-provoking situations while practicing coping strategies in a secure, controlled VE. Moreover, IVR-CBT provides a supportive learning environment that allows users to experiment with alternative behaviors in a step-by-step manner, thereby enabling cognitive restructuring and learning of suitable behavioral responses to SUD-related symptoms. This aligns with the core objectives for SUDs in standard addiction care treatments that are based on CBT principles [27], including psychoeducation, skill development for coping strategies, developing more adaptive thinking patterns, strengthening problem-solving skills in a practical manner, and relapse prevention through self-control and reinforcement of positive behaviors [28]. However, research in interdisciplinary teams is needed to understand design requirements to develop IVR-CBT interventions, as well as implementation into treatment as usual.

This study reports on 2 explorative studies conducted with patients in a specialized addiction clinic for individuals with MBID and SUD. Study 1 aimed to identify patient-reported high-risk situations and craving triggers relevant to alcohol use during preparation for temporary clinical leave. Study 2 aimed to explore the feasibility and acceptability of engaging patients with MBID in an IVR protocol that induces nicotine craving while embedding coping components in virtual reality (VR; eg, distraction, relaxation, and physical activity). Feasibility was defined as completion of IVR phases and tolerability (eg, need for support/breaks, discontinuation), and acceptability was defined as perceived realism and perceived usefulness. Given the substance-specific focus of each study, findings are interpreted as exploratory and design-oriented, without assuming transferability of identified triggers or coping preferences across substances. Across both studies, our overarching goal was to generate initial design requirements and clinical integration considerations for future IVR-CBT modules for MBID and SUD populations.

## Methods

### Study 1: Identifying High-Risk Situations and Triggers for IVR-CBT

#### Study Design and Participants

We explored high-risk situations and triggers to prepare patients with MBID and alcohol use disorder (AUD) for temporary clinical leave during treatment as usual. In total, 10 adults with MBID, receiving inpatient AUD therapy, were included using convenience sampling. Participants were recruited by their treating therapist within the inpatient clinic when they met the study criteria and were considered able to participate. Diagnostics for MBID were conducted by psychologists in the addiction clinic or by other medical institutions in the care chain of patients (eg, disability care). Exclusion criteria included severe psychiatric disorders (eg, psychosis) or active substance use. Eight of ten patients had previous experience with IVR, mainly via participation in the previous studies by our research group. Data were collected from January to April in 2021 in a Dutch addiction clinic specialized in the treatment of patients with MBID and SUD.

#### Ethical Considerations

This research was approved by the Saxion University of Applied Sciences in Enschede, as well as the scientific board of Tactus Addiction Care (OZP 26-112020). Personal identifiable information was archived separately from research data at Tactus Addiction Care to protect our patients from data breaches. Moreover, we pseudonymized data and deleted related audio recordings after transcription. Before starting the interviews, participants signed an informed consent. Patients received no compensation for participation.

#### Materials and Procedures

We conducted semistructured interviews with 7 open questions to explore (1) how patients would prefer to use IVR for practicing the clinical leave (2 questions), (2) which alcohol-related situations (1 question) induce alcohol craving, and (3) which triggers (1 question) induce alcohol craving. The interview guide (Multimedia Appendix 1) was pilot tested with 2 patients prior to data collection. The researcher explained the study procedure by using examples (eg, photos and videos) from the “Go up in smoke” project [13] to familiarize patients unfamiliar with IVR with relevant concepts. The “Go up in smoke” project focuses on the induction of nicotine craving using triggers and the reduction of craving using coping skills in IVR. Subsequently, semistructured interviews were held in a quiet office in the addiction clinic and were audio recorded.

#### Data Analysis

The audio files were transcribed verbatim and analyzed based on Braun and Clarke’s reflexive thematic analysis approach [29,30]. For this, the researcher (Berlind van Ast) followed the 6-step protocol: for (1) data familiarization, the researchers listened to the recordings and read the transcripts. Subsequently, (2) relevant segments in the data were identified, coded, and collated. Then, (3) initial themes were generated using the coded data and (4) reviewed by revisiting themes with respect to the aims. Finally, (5) themes were defined, and the (6) report was produced. For this, we used the qualitative data analysis program Atlas.ti (v.9; ATLAS.ti Scientific Software Development GmbH).

During this process, researchers (Berlind van Ast and Jolien Jongeling) reflected together on themes generated during the 6-step analysis (steps 4 and 5), as well as during meetings with supervisors. Both researchers hold a degree in advanced nursing studies and were working in the addiction clinics at the time of data collection. Therefore, both researchers possess previous knowledge about the target group, as well as skills to engage in research with the MBID population. However, previous experiences with addiction care and the context of this research focusing on the IVR-CBT paradigm may influence the confirmability and transferability of findings.

### Study 2: (Don’t) Go Up in Smoke, Exploring Coping Strategies to Reduce Nicotine Craving in IVR

#### Study Design and Participants

In study 2, we implemented and evaluated coping skills in IVR that were derived from self-control techniques (eg, distance, distraction, and declare) that are taught during the less booze or drugs [9] treatment for patients with comorbid MBID. For this, we explored typical risk-coping scenarios in IVR to reduce nicotine craving in a convenience sample of 10 non–nicotine-deprived adults with MBID and nicotine dependence (ND) (Fagerström ≥5) undergoing inpatient treatment in a Dutch addiction clinic. Participants were recruited by their treating therapist when they met the study criteria and were considered able to participate. Diagnostics for MBID were conducted by psychologists in the addiction clinic or by other medical institutions in the care chain of patients (eg, disability care). Exclusion criteria included a history of migraine, epilepsy, motion sickness, severe psychiatric disorder (eg, psychosis), and use of nicotine replacement therapy to protect participants from IVR-induced symptoms (eg, cybersickness and hallucination) induced by the IVR device or, in the case of nicotine replacement therapies, to avoid introducing bias on the data collected. Only one of the 10 patients had prior experience with IVR. Data were collected from January to April 2021 in a Dutch addiction clinic specialized in the treatment of patients with MBID and SUD.

#### Ethical Considerations

Ethical approval as nonmedical research was given by the medical ethics board of the MST hospital in Enschede (K19-34). Moreover, this work was approved by the scientific board of Tactus Addiction Care. Personal identifiable information was archived separately from research data at Tactus Addiction Care to protect our vulnerable patients from data breaches. Moreover, we pseudonymized data and deleted related audio recordings after transcription.

All participants were welcomed by the researcher and thoroughly informed about the research procedure. After informed consent was obtained, the audio and screen recordings were started. The participants received no compensation for participation.

#### Measures

ND was assessed with the Dutch version of the Fagerström Test for nicotine dependence (FTND) [31]. The FTND is a 6-item questionnaire assessing ND severity with scores ranging from 0 to 10. Scores of 0‐2 indicate a light dependence, 3‐5 a moderate dependence, 6‐7 a severe dependence, and 8‐10 a very severe dependence.

Nicotine craving was assessed with the Questionnaire of Smoking Urges Brief (QSU-Brief) and by using a Visual Analog Scale (VAS). The QSU-Brief is a 10-item questionnaire assessing the urge to smoke [32]. Scores on each item range from 1 (“Strongly disagree”) to 7 (“Strongly agree”). The total score is obtained by calculating the mean of the 10 items. The VAS is a single-item scale ranging from 0 to 10, where 0 is interpreted as “no craving” and 10 as “severe craving.”

A semistructured interview (Multimedia Appendix 2) with 14 open questions was conducted to explore (1) VE to induce and reduce nicotine craving (3 questions), (2) explored coping strategies to reduce nicotine craving (5 questions) and alternatives in IVR to reduce nicotine craving (5 questions), and (3) potential applications of IVR during treatment (1 question).

#### Hardware and VE

We used an HTC VIVE Pro Eye HMD (HTC Corp), 1440×1600 pixels per eye (2880×1600 combined), a 90 Hz refresh rate, and an 110-degree field of view, base stations, controllers, and a compatible laptop to display the IVR to the patient.

**Figure 1.**
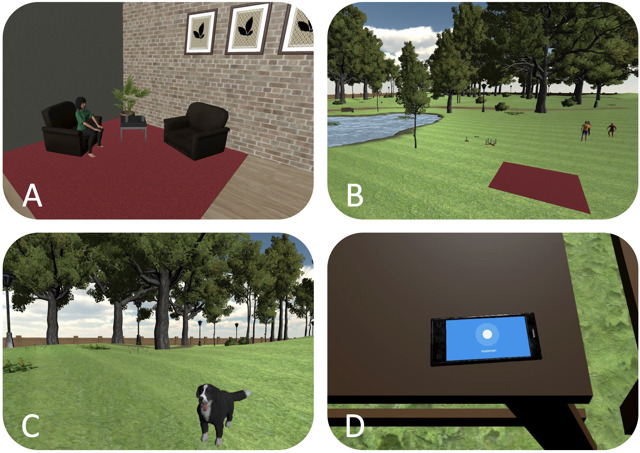
The virtual environment (VE) for craving reduction. (A) Mindfulness via a virtual therapist, (B) Park with sporting virtual humans, relaxation areas, and games, (C) Walk in the park with a dog, and (D) Breathing exercises on a virtual smartphone.

The VE was developed in Unity3D (v.2019.2.3f1; Unity Technologies) using the SteamVR Software Development Kit (Valve Corporation) and was partially adapted from previous work by our department [13], containing two main scenarios: (1) craving induction and (2) reduction (see Figures 1A-1D). The craving induction scenario comprised 3 main areas [13], including a crossroad with a bus stop, at home with a garden, and a restaurant with a terrace and smoking goods vendor. In contrast, the craving reduction scenario comprised 2 environments, including a therapy room with 2 virtual humans guiding 2 different mindfulness exercises that were prerecorded with a psychomotor therapist, as well as a virtual cat in the front yard, a park with a lake, a walkway, sporting agents, a blanket with calming music, a ring toss game, a badminton set, a dog that can be taken for a walk, and benches with a virtual smartphone to conduct a visual breathing exercise. Audio cues respecting the restaurant, interactive humans, cigarettes, blowing wind, running water, and calming music were used to deepen immersion. Participants were able to use teleport locomotion and an approximately 2×2 m room-scale area for natural locomotion.

**Figure 2.**
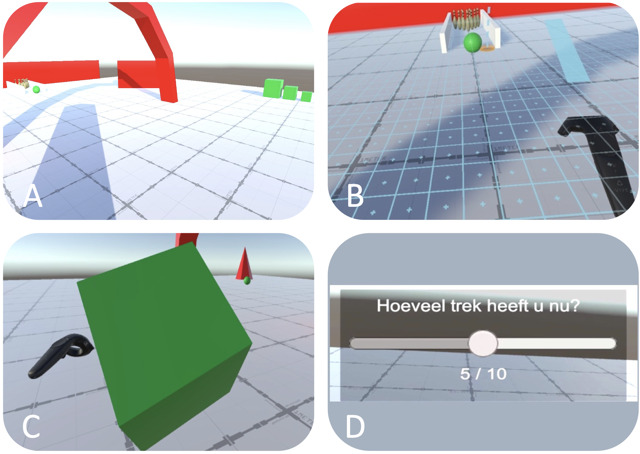
The tutorial environment to train interactions in immersive virtual reality (IVR), including a (A) “Walking track” to train teleporting, (B) “Bowling alley” to grab and throw a bowling ball, (C) three interactable cubes of different sizes to be stacked, and (D) Visual Analog Scale (VAS) for craving assessments.

To train patients in using the controllers and ensure habitation within the IVR, a neutral tutorial was used with specific interactions (ie, grabbing objects or teleporting) by using a plane with a walking track, 3 interactable cubes, and a bowling alley (see Figure 2A-2D).

#### Procedure

The research was carried out in a quiet room in an addiction clinic to enhance an ecologically valid surrounding that replicated the usual treatment setting. The procedure (maximum 90 min) was divided into five phases: (1) preparation (≈15 min), (2) tutorial (≈10 min), (3) trigger-induced craving (≈15 min), (4) coping skills and craving reduction exploration (≈15 min), as well as (5) postquestionnaires (≈30 min). During the preparation, demographics and baseline characteristics (ie, FTND and QSU-Brief) were assessed. If necessary, patients were allowed small breaks during phases 1‐5 without leaving the room (eg, to smoke). At the beginning of the sessions, participants engaged in a short tutorial session in a neutral VE to become familiar with the HMD and controls and rate their craving using the VAS. Upon completion, participants removed the HMD and were verbally asked the QSU-Brief again before entering the cue reactivity environment. Participants were asked to wear the HMD again and conduct a guided exploration (see [13] of 5 specific situations aiming to induce cravings, including bus stop, restaurant terrace, a vendor with smoking goods, an at-home environment with a garden, and a man sitting on a bench smoking). After completing the craving induction stage, patients were instructed to interact with various coping skills, including distraction (ie, breathing and mindfulness exercises, sport, relaxation, and animals), distance (ie, walk in park or go outside), different thinking, different acting (ie, say “no” when a cigarette is offered, drink a soft drink, or use a nicotine patch), declaring (ie, use a virtual phone to call for help), which were derived from existing therapy protocols for people with dual diagnosis [9]. During both phases, patients were asked to rate their cravings after each interaction using the in-game VAS, followed by removing the HMD and completing the QSU-Brief. Finally, the semistructured interview was conducted verbally with participants. During the use of the HMD, patients’ therapists were close by to provide help if needed.

#### Data Analysis

The audio files were transcribed verbatim and analyzed based on Braun and Clarke’s reflexive thematic analysis approach [29,30]. For (1) data familiarization, the researcher (Jolien Jongeling) listened to the recording and read the transcript. Then, (2) relevant segments were identified, coded, and collated. The (3) initial themes were generated using the coded data and (4) reviewed by revisiting themes with respect to the dataset and goal. Finally, (5) the themes were defined, and the (6) report was produced. For this, we used the qualitative data analysis program Atlas.ti (v. 9). Data from the QSU-Brief and VAS were analyzed descriptively using RStudio (v. 1.3.1093; PBC).

During this process, researchers (ie, Jolien Jongeling and Berlind van Ast) reflected together on themes generated during the 6-step analysis (steps 4 and 5), as well as during meetings with supervisors (ie, SL and JV). Both researchers pursued a master’s degree in advanced nursing studies and worked in the addiction clinic described in both studies. Therefore, both researchers possess previous knowledge about our target group, as well as skills to engage in research with the MBID population. However, previous professional experiences and the context of this research with a focus on the IVR-CBT paradigm may influence the confirmability and transferability of findings to other settings.

## Results

### Study 1. Identifying High-Risk Situations and Triggers for IVR-CBT

#### Sample Description

Participants (n=10) had a mean age of 40.5 (9.7) years, and most identified as female (n=6). The sample included patients with BIF (n=7) and MID (n=3). Notably, most participants (n=7) had prior IVR experience from the “Go up in smoke” project.

#### High-Risk Situations and Triggers That Elicit Alcohol Craving

The participants identified high-risk situations (see Table 1), including at home, supermarkets, social gatherings, and being outside.

In the home environment, participants mostly described the living room to elicit alcohol habits, though these situations are usually individual, as others describe the bathroom or kitchen. Also, the conditions vary from clean to messy (eg, with bottles all around). Regarding the supermarket, participants described associations and confrontation with alcohol cues (eg, advertisement), potentially triggering affective states (“Then my craving goes up and I start sweating and then I just get difficult thoughts. That I have to drink. That everything gets better when you drink. Yes, very nervous, stressful.” [P02]). Social gatherings include meetings with families and peers that entail invitations to drink, including peer pressure and conflict situations (“It is when I am with my father, that he triggers me with his negative energy, my living room, and the supermarket.” [P1]). Finally, being outside passing beer gardens, supermarkets, or being in public transport (ie, bus and train) is probably causing distress or invitations from others when walking by.

**Table 1.** High-risk situations and triggers in patients with mild to borderline intellectual disability (MBID) and alcohol use disorder (AUD).

Theme	Example
High-risk situations	
At home	“As soon as I enter, I sit on the couch, grab a glass and turn on the football. Then I just sit and consume it on my own.” (P08)
Supermarket	“You enter the store and then you see, and you are confronted and then you feel that urge for alcohol. And even if I were to walk by, I would still see them, and they would look at me too. They look like: ‘don't forget me.’*”* (P08)
Social gatherings	“Often you will meet someone you know, and they often know that you like a glass of wine and a beer. And then it’s: ‘hey, come and sit down, nice and cosy.’” (P07)
Being outside	“Put [Dutch city] in there. There they have to do it themselves. Either by bus or by train. You could combine that with those virtual reality glasses, with the person who either has to go by bus or by train and see how the person reacts.” (P01)
Triggers	
Multisensory experiences	“I also deliberately don’t go through that hallway anymore, but you see the offer when you come in these days and it’s actually terrible, like they do it on the TV.” (P05)
Social influences	“For example, if someone would like to belong to a group, then that group encourages: ‘Hey, join us!’ You should put something like that in it. I would experience that as a trigger.” (P07)
Affective states	“When I’m high in tension, I tend to go to the store very quickly. Then I’ll go get some alcohol to push that away again. To feel relaxed again.*”* (P01)
Personal habits	“I associate money with alcohol because then these are my groceries.” (P08)

Triggers (see Table 1) to use alcohol in the abovementioned high-risk settings can be classified into multisensory experiences, social influences, affective states, and personal habits. The participants described seeing, smelling, and hearing alcohol-related cues in daily situations, such as people drinking, (alcohol) advertisements, bottles, supermarkets, soccer on TV (with sound), and (Dutch) music during social gatherings. Social influences comprise the described invitations, pressure, and conflict situations with (relevant) others to elicit strong distress/cravings. Generally, participants described a spectrum of affective states, such as distress, boredom, and euphoria as causes of alcohol craving. Finally, personal habits, such as rewarding oneself, nice weather, watching TV, having money in one’s wallet, or completing work, were also described, often intertwined with the aforementioned triggers (ie, multisensory cues, social influences, and affective states).

#### Applications of IVR for CBT in Clinical Care

The preferred use of IVR during clinical care in patients with MBID and AUD can be divided into assessment and treatment (see Table 2).

**Table 2.** Preferred use of immersive virtual reality (IVR) for cognitive behavioral therapy (CBT) in patients with mild to borderline intellectual disability (MBID) and alcohol use disorder (AUD).

Theme	Example
Assessment	
Identify high-risk situations and triggers	“I will feel safe. Yes, because suppose you really get those craving moments or a panic attack, then you have a backup to rely on.” (P01)
Coping skills training	
Discuss feelings during high-risk situations with therapists	“Because you are still in a safe environment, you can better discuss what you are feeling at that moment and how you can best deal with it.” (P07)
Training of distraction techniques for coping with alcohol craving	*“*Practicing with such glasses to look for distraction, that is very important. (.) For example, that I get a trigger to take a walk. So, I didn’t do that before, I just remained seated and then went for a walk to the fridge.” (P06)
Learn to say “no” when offered alcohol	*“*Seeing other people drinking seems like a very good thing to practice [with IVR], you will soon encounter that at birthdays, and I just want to be able to do it without it. And if they offer me one then I want to be stronger to say ‘no.’” (P05)

For assessment, participants reported the need to identify personal risk situations and triggers using IVR. This includes measuring craving in the given context (by using a VAS) to gain insights into one’s own vulnerabilities. Regarding treatment, participants reported the wish to discuss feelings during risk situations with therapists, to train distraction techniques for coping with cue reactivity, and learn to say “no” when offered alcohol, for example, by choosing alternatives. In doing so, participants said they would like to train and rehearse repeatedly before leaving the clinic (“If you have always said ‘yes’ [to substance use] it is very strange for you to say ‘no.’ And I think the more you practice that, the easier it gets.” [P07]). Most participants indicated a positive intention to use IVR for practicing clinical leave (“Yes, that would be nice, because then you are not immediately placed in the real situation, and you can learn to deal with your feelings and with your craving.” [P02]), though some found it redundant or anxiety-evoking (“It was too scary for me. Everything overwhelms you, so to speak.” [P10]).

### Study 2: (Don’t) Go Up in Smoke–Exploring Coping Strategies to Reduce Nicotine Craving in IVR

#### Sample Description

Participants (n=10) had a mean age of 39 (9.0) years, of which half (n=5) identified as female. The sample included patients with BIF (n=7) and MID (n=3). The FTND was moderate (n=2), severe (n=4), and very severe (n=4), respectively. Patients smoked on average 18.7 (4.2) per day, and most (n=9) had no prior IVR experience.

#### IVR Craving Induction and Procedural Remarks

All participants reported the IVR to be craving-inducing. Especially, the man offering a cigarette and the bus stop were mentioned to cause severe cravings (“Yes and that man on that bench, who then offers you a cigarette and at that moment you just notice something goes through you, oh yes delicious.” [S08]). Though some participants reported prior to using the IVR worries about their abilities to navigate and interact, the researchers observed mostly positive remarks after immersion in IVR. However, 2 patients described anxiety issues related to using IVR (“I do find it a little scary” [S01]), resulting in a single dropout.

#### Coping Strategies to Reduce Nicotine Craving in IVR

For the implemented coping skills (see Table 3), participants named distraction through games and animals; relaxation (ie, mindfulness and breathing exercises); and physical activity (ie, sport going and for a walk in the park [with a virtual dog]) as coping strategies to reduce nicotine craving in IVR. Moreover, all participants engaged in mindfulness/breathing exercises delivered by virtual humans or smartphones. The strategies were described as appealing, though some described control and concentration troubles (“Then I have to be very quiet. No people around me, because then it won’t work.” [S10]). Furthermore, engaging in physical activity was pleasant, for instance, through sports and games (eg, bowling, throwing rings, and stacking cubes), as most participants mentioned the neutral tutorial to reduce craving (“I immediately stopped thinking about smoking” [S06]). For animals, people enjoyed petting and walking a virtual dog and playing with a virtual cat, as some missed their pets during their clinical stay. Noteworthy, being busy with IVR was described as distracting by many participants. Coping strategies to add to IVR include yoga, additional sports (eg, football and basketball), meditation, talking to a therapist or other patients, and creative tasks.

**Table 3.** Coping strategies to reduce nicotine craving in immersive virtual reality (IVR).

Theme	Example
Distraction	
Interactive games (eg, bowling, ring toss, and stacking cubes)	“But that’s because I focused on the actions I had to do, that I focused very much on that actually, so I might actually suppress the urge […].” (S05)
Virtual pets	“But also with my cat, which walks with me to the store, that sort of thing, yes crazy. Yes, I miss that animal so much and then they say it’s just a cat. Yeah, it’s easy for you to say. I see him as my child.” (S10).
Sitting close to water (on blanket hearing music)	“That you were distracted, I think, especially that last one beside the water and then with that dog.” (S02)
Talking to therapist or other patients^a^	“[…] that someone tells you that the craving will go away and so on, that you will think about it, that you maybe can let it go a bit better.” (S08)
Creative tasks	“The open air, the bench to sit down quietly and eh meditate a bit or if necessary, bring a coloring book, because I still like to color very much.” (S05)
Relaxation	
Mindfulness	“It did diminish a little with the relaxation exercises, when I sat in that chair like that for a while.” (S08)
Breathing exercises	“[…] of breathing in and out, that’s actually a kind of relaxation exercise.” (S05)
Physical activity	
Going for a walk in the park (with a virtual dog)	“It was more feeling good again, that dog came running to me and eh you miss that very much here” (S08)
Sports (eg, squats, yoga, basketball, football)	*“*[…] because it is anyway necessary for my fitness, but also for distraction and yes sport is just better on all fronts.” (S04)

a Not implemented in our immersive virtual reality coping strategies exploration for craving reduction.

#### Subjective Craving During Baseline, Tutorial, Craving Induction, and Craving Reduction

Table 4 describes data obtained from the QSU-Brief and VAS across the different measures at baseline (T0), tutorial (T1), craving induction (T2), and craving reduction (T3). The results show tendencies for craving reduction during the tutorial (T1) and coping skills exploration (T3).

**Table 4.** Difference in craving on Questionnaire on Smoking Urges Brief (QSU-Brief) and Visual Analogue Scale (VAS) at T0-T3.

Measures	T0	T1	T2	T3
QSU-Brief^a^				
Mean (SD)	3.41 (1.1)	3.04 (1.4)	4.20 (1.5)	3.66 (1.2)
Median (IQR)	3.50 (3.0-3.8)	3.15 (1.6-4.3)	4.35 (3.5-5.2)	3.55 (3.0-4.3)
VAS^b^ in IVR^c^				
Mean (SD)	4.22 (2.2)	2.86 (2.8)	5.34 (1.8)	2.50 (2.1)
Median (IQR)	4.00 (4.0-5.0)	2.00 (1.0-4.0)	5.57 (4.6-6.6)	2.00 (1.5-2.8)

a QSU-Brief: Questionnaire on Smoking Urges Brief.

b VAS: Visual Analogue Scale.

c IVR: immersive virtual reality.

## Discussion

### Principal Findings

This study reported 2 exploratory, design-oriented studies that were conducted in an inpatient MBID setting, examining high-risk situations, triggers, and coping strategies to inform the development and clinical integration of IVR-supported elements for substance-use treatment.

In the first study, qualitative interviews were used to identify alcohol-related high-risk situations, triggers, and patient-defined treatment goals in preparation for temporary clinical leave. Participants identified a range of high-risk situations for AUD, including being at home, in supermarkets, at social gatherings, in public spaces, or while traveling. Alcohol craving in these contexts was associated with multiple types of triggers, including multisensory cues, social influences, affective states, and personal habits. Participants described these alcohol-related high-risk situations as challenging and expressed interest in using IVR to identify personal triggers in context, discuss emotional responses while being immersed, and repeatedly rehearse refusal skills or alternative actions for temporary clinical leave.

In the second study, an existing IVR cue reactivity environment was used to explore the feasibility and acceptability of practicing coping strategies for nicotine craving, alongside descriptive craving assessments. All participants reported that the IVR elicited nicotine craving, and most were able to complete the procedure. This suggests that the use of IVR was feasible in an inpatient MBID setting, when therapist support was available. Participants perceived several coping strategies as helpful, which were related to distraction (eg, games and virtual pets), relaxation (eg, mindfulness and breathing exercises), and physical activity (eg, sport and walking the dog). Descriptively, nicotine craving tended to increase during cue exposure and decrease during the tutorial and coping phases. Notably, participants differed in how they experienced the specific IVR scenarios, and some required additional support due to anxiety or unfamiliarity with IVR. For example, one participant described a virtual riverside environment as triggering for nicotine craving due to personal associations with alcohol use (“I live close to the IJssel [Dutch river] myself and that was a very triggering place for me last summer, because automatically people sit there to drink alcohol*”* [S04]), whereas other participants experienced similar scenarios as craving reducing. This illustrates that the subjective meaning of IVR scenarios can vary substantially between individuals and that certain environments may evoke unintended craving responses depending on personal history.

Taken together, these findings suggest that IVR may provide a structured and experiential format for identifying high-risk situations and rehearsing coping responses in context for individuals with MBID. This is particularly relevant given the barriers in MBID treatment, including difficulties with abstract reflection and transfer of coping skills to everyday environments [2,8].

### Interpretation and Comparison With Prior Work

Participants in Study 1 identified different high-risk alcohol situations such as supermarkets, home settings, social gatherings, and public spaces. These findings are consistent with previous research on triggers in AUD, which has highlighted the role of environmental cues such as bars and stores in eliciting cravings [21,33]. However, participants in the present study frequently described these environments in combination with social influences (eg, peer pressure and interpersonal conflict) and affective states (eg, tension, negative affect, and distress) as triggers for alcohol craving. For example, in supermarkets, alcohol exposure was described as stressful and confrontational due to advertisements and visibility of alcohol products. Social gatherings were linked to invitations and pressure to drink. In the home setting, alcohol use seemed to be described as embedded in a habitual sequence of actions, such as coming home, sitting on the couch, watching football, and then drinking.

These findings indicate that alcohol craving in this MBID sample was experienced as embedded in daily routines and interpersonal situations, rather than triggered by exposure to environmental stimuli alone. This aligns with broader conceptualizations of craving as context-dependent while being influenced by affective and social processes [33]. In this study, participants described complex situations in which contextual cues, emotions, and social interactions were closely intertwined. For individuals with MBID, who often find it difficult to analyze and describe their everyday risk situations [2,8], addressing such interconnected triggers in a traditional CBT session can be challenging. CBT typically relies on retrospective discussion and functional analysis of high-risk situations. This requires patients to reconstruct the situation, identify the links between context, emotion, and behavior and consider alternative responses. Within this context, IVR may offer a complementary way to examine and rehearse high-risk situations in a more concrete and structured way.

While most IVR cue reactivity research operationalizes craving through predefined substance-use environments, such as bars or public venues [10,17,18], the present study identified high-risk situations and triggers for alcohol craving in patients with MBID through a bottom-up approach derived directly via interviews with patients. The situations described by participants were often routine and home-based and embedded in everyday activities rather than limited to prototypical drinking locations. Using these patient-derived situations as a starting point when developing IVR scenarios may increase the practical relevance of IVR within MBID inpatient treatment.

In the second study, participants explored several coping strategies to reduce nicotine craving in IVR. All patients reported that the IVR environment induced nicotine craving, confirming its ecological validity, consistent with earlier findings [13]. Interestingly, nicotine craving decreased during the tutorial phase, which contrasts with previous results that showed increased nicotine craving during immersion [13]. One possible explanation for this difference is that the gamified tutorial required sustained attention and functioned as a distraction, thereby reducing nicotine craving. This observation suggests that the structure and content of the tutorial phase may influence craving responses and should therefore be considered carefully when designing IVR protocols.

Participants perceived coping strategies that were embedded within IVR as helpful, particularly those that involved concrete actions such as interacting with virtual animals or engaging in games. This suggests that including active coping components within IVR may be important, rather than relying on cue exposure alone [22,26]. However, participants differed in how they experienced certain environments, which highlights the need for careful scenario selection and therapist guidance. The riverside example illustrates that environments intended to be calming may evoke craving if they are associated with prior substance use experiences. This underscores the importance of assessing personal associations and monitoring tolerability when integrating IVR into clinical care.

### Implications for the Development of an IVR-CBT for Patients With MBID

The findings from both studies provide practical starting points for the development of IVR-supported CBT elements within MBID SUD treatment. The present studies identified high-risk situations and coping strategies that patients themselves considered as relevant within inpatient MBID treatment and demonstrated that practicing coping skills within IVR was feasible and acceptable when therapist support was available. Participants also described treatment-related goals, such as discussing feelings with therapists during IVR sessions, practicing refusal skills, and learning to manage craving-related distress. These goals align with CBT mechanisms such as functional analysis and coping skills training [34], as well as with MBID-adapted CBT protocols that are used within Dutch inpatient treatment settings at the time of this writing [9].

Although clinical effectiveness was not evaluated, the descriptive reductions in craving during the coping phases suggest that CBT-based coping strategies, including self-control techniques tailored to the needs of patients with MBID, can be operationalized within IVR environments. The possibility to simulate socially and emotionally complex situations in a structured and controlled setting may allow for repeated rehearsal, which can be difficult to stimulate in traditional therapy but can be practiced safely and repeatedly in IVR. Prior studies have shown that IVR is effective at establishing cue reactivity by simulating real-life scenarios related to substance use. However, many applications focused primarily on exposure to SUD-related cues, without embedding active coping skill training or other therapeutic elements that are essential for behavioral change [35]. Integrating coping rehearsal directly within IVR may help to address this limitation by combining immersive exposure with the immediate practice of coping strategies.

Based on the findings from both studies, several design and implementation considerations for developing IVR-CBT interventions (ie, trigger-coping scenarios) can be identified. First, IVR environments may be useful for supporting two fundamental CBT objectives: (1) identification of high-risk situations and triggers (CBT: stimulus control) and (2) coping with trigger-induced craving (CBT: stimulus response prevention) [36]. IVR may function as a complementary modality within existing CBT protocols by enabling in-context rehearsal of skills that are otherwise discussed retrospectively. In doing so, a structured IVR-CBT framework may be developed that systematically links CBT objectives to certain IVR training modules with risk-coping scenarios, grounded in both CBT theory and immersive learning design. Stimulus control could be addressed through specific IVR trigger-assessment modules, in which patients, for instance, identify, label, and rate high-risk situations or triggers via simplified interfaces and real-time feedback tools via either the IVR or the therapist. Once patients can recognize and assess their high-risk situations and triggers, the next step would involve training them to actively manage their responses, thereby moving from stimulus control to stimulus-response prevention. The transition from stimulus control to stimulus-response prevention can be supported by an IVR-CBT framework with a staged approach that aligns technological affordances with a pedagogical learning design, as recommended by [37], by sequencing skill acquisition through concrete, repetitive, and context-specific activities.

Moreover, given the variability in individual trigger experiences observed in this study, IVR systems may benefit from allowing flexibility in scenario selection and coping options. Personalization through therapist-patient goal setting [37] and the use of customized scenarios that are based on each patient’s individual triggers and coping needs [38] might enhance clinical relevance. For instance, patients presenting with predominantly social triggers can, for instance, engage in peer-pressure simulations involving assertive refusal training, while patients with predominantly environmental triggers (eg, being in a bar) may work within location-based scenarios that focus more on stimulus control and rehearsal of coping strategies (eg, going for a walk). However, it is still uncertain whether individualized tailoring improves outcomes when compared to standardized IVR modules, as this still requires empirical evaluation.

Furthermore, IVR content may benefit from alignment with individualized high-risk situations and trigger profiles systematically linked to personalized coping strategies, while keeping a balance between personalized IVRs and standardized treatments [39]. It is important that researchers distinguish therapeutic mechanisms from educational ones, as skill learning in IVR may occur even in the absence of immediate relief effects, which are typically associated with real-world coping. Therefore, future studies should not only evaluate whether patients can learn coping strategies in IVR but also how these strategies generalize to behavior outside of IVR. Finally, the development of IVR-CBT protocols may benefit from interdisciplinary collaboration to address clinical, technological, and implementation considerations, including questions related to intensity and dosage. Future research could contribute to the development and empirical evaluation of a CBT-informed framework for IVR interventions that links identified triggers to coping strategies across structured learning stages. In addition, further studies should examine personalization capabilities, implementation barriers, and acceptability of IVR-CBT across broader MBID populations and diverse treatment settings.

### Limitations

Several limitations should be considered. First, both studies included small convenience samples recruited from a single clinic; therefore, limiting the extent to which the results can be generalized to other settings or MBID populations. Our convenience sampling might have over-represented individuals with MBID who were comparatively stable, motivated, and comfortable with technology since participation in the studies was voluntary and patients with severe psychiatric instability were excluded, resulting in selection bias. As a result, our findings may not fully apply to the perspectives and needs of individuals with higher levels of psychiatric complexity or lower technological literacy. Second, the 2 studies focused on different substances and different therapeutic targets. While AUD and ND may share some features, they may also differ in contextual triggers, behavioral patterns, and treatment mechanisms; therefore, the findings should be interpreted within each specific substance context. While overlapping processes such as cue reactivity and coping rehearsal may be relevant across SUDs, the specific triggers and coping elements that were identified in this work are substance-specific and design-oriented. In addition, the studies were exploratory and design-oriented, thereby not assessing clinical effectiveness, long-term outcomes, or the generalization of learned coping strategies beyond the IVR context. Third, the data were collected verbally by nurses (in training to become specialists) to resemble a common treatment setting. However, this might have increased social desirability bias (eg, see contrary craving effects during the tutorial phase), though paper-based assessments appear too complex for our group. Fourth, the interactions within the IVR were limited and of short duration due to the explorative nature of our study. This might have restricted the patient’s opportunity to fully practice coping responses extensively and may have limited the extent to which craving reduction could be observed. Fifth, it is important to note that we demonstrated and explored coping skills by using a pre-existing IVR system. This may have caused confirmation bias because participants’ given responses may have been influenced by the available specific examples and functionalities that were presented, which may have limited the identification of additional coping strategies. Instead, our explorative research aimed to generate or understand how IVR can be integrated into current CBT treatment in patients with MBID and SUD. Finally, participants might have participated in prior studies by our group, which could influence the data collected.

### Conclusions

IVR-supported CBT elements appear feasible and acceptable in an inpatient MBID setting when appropriate support is available. The present studies provide concrete, patient-derived design insights into alcohol-related high-risk situations and nicotine-related coping elements that can be embedded in IVR. The findings of these studies suggest that IVR may help to reduce barriers that are associated with abstract verbal reflection by enabling in-context identification of triggers and repeated rehearsal of coping responses within a controlled environment. However, IVR environments may activate personally learned substance-use associations that differ between individuals. Therefore, careful scenario selection, real-time craving monitoring, and therapist guidance seem to be necessary to prevent unintended increases in craving.

## Supplementary material

10.2196/82601 Multimedia Appendix 1 Semistructured interview on alcohol-related high-risk situations and triggers for immersive virtual reality-cognitive behavioral therapy (IVR-CBT).

10.2196/82601 Multimedia Appendix 2 Semistructured interview on coping strategies to reduce nicotine craving in immersive virtual reality (IVR).

## Abbreviations

AUD: alcohol use disorder
BIF: borderline intellectual functioning
CBT: cognitive behavioral therapy
FTND: Fagerström Test for Nicotine Dependence
HMD: head-mounted display
IVR: immersive virtual reality
MBID: mild to borderline intellectual disability
MID: mild intellectual disability
ND: nicotine dependence
QSU-Brief: Questionnaire of Smoking Urges-Brief
SUD: substance use disorder
VAS: Visual Analogue Scale
VE: virtual environment
VR: virtual reality
VRET: virtual reality exposure therapy
