# Supplementary material for: Immersive Virtual Reality–Supported Cognitive-Behavioral Therapy for Patients With Mild to Borderline Intellectual Disabilities and Substance Use Disorders: Two Exploratory Studies
Source: JMIR XR Spat Comput. 2026 May 4;3:e82601. doi: 10.2196/82601 (PMC13202510; doi:10.2196/82601)
Supplement: Multimedia Appendix 1 [file xr-v3-e82601-s001.docx]

| **Topic** | **Question** |
| --- | --- |
| Practicing clinical leave using VR | 1. What do you think about practicing clinical leave using a VR headset? |
| High-risk situations during leave | 1. Which difficult leave situation (e.g. where you might relapse) would you want to practice?   Follow up:   - 1. What happens in such a difficult situation?   2. What does that environment look like you?   3. What else is present there?   4. Who is present there? |
| Alcohol-related triggers | 1. What gives you the urge to drink alcohol?   Follow up:   - 1. What type of drink?   2. Which brand?   3. What sounds?   4. What smells?   5. Which days?   6. What times?   7. What people? (e.g. seeing old drinking buddies, social pressure, seeing drinking, being invited to drink)   8. What mood or state of mind?   9. Are there other things that trigger your urge to drink? |
| Use during treatment | 1. How would you prefer to practice for leave with a VR headset?   Follow up:   - 1. Practice the 6 D's (distance, distraction, different thinking – different acting, declare, deals, doing great)?   2. Measure craving?   3. Other? |
