# Supplementary material for: Immersive Virtual Reality–Supported Cognitive-Behavioral Therapy for Patients With Mild to Borderline Intellectual Disabilities and Substance Use Disorders: Two Exploratory Studies
Source: JMIR XR Spat Comput. 2026 May 4;3:e82601. doi: 10.2196/82601 (PMC13202510; doi:10.2196/82601)
Supplement: Multimedia Appendix 2 [file xr-v3-e82601-s002.docx]

| **Topic** | **Question** |
| --- | --- |
| Opening question | 1. How did you experience going through this virtual environment? |
| Virtual environment | 1. Was there anything in the environment that gave you the urge to smoke? If so, what? 2. Did you miss anything in this environment? If so, what? |
| Coping techniques | 1. What did you choose in the virtual world? 2. Why did you choose these techniques? 3. What do you think about the chosen techniques? 4. What would you like to practice more within virtual reality? 5. What else could help you? |
| Alternatives | 1. Which environment helped you find distraction? 2. Did you recognize anything from your addiction treatment? Explain. 3. What did you recognize? 4. What would you like to be able to do/feel more? 5. Would it help you if you received a reward within this VR for not smoking? |
| Closing question | 1. Do you think practicing with virtual reality would help you quit smoking? Why or why not? Other remarks? |
